# Population Bottlenecks and Intra-host Evolution During Human-to-Human Transmission of SARS-CoV-2

**DOI:** 10.3389/fmed.2021.585358

**Published:** 2021-02-15

**Authors:** Daxi Wang, Yanqun Wang, Wanying Sun, Lu Zhang, Jingkai Ji, Zhaoyong Zhang, Xinyi Cheng, Yimin Li, Fei Xiao, Airu Zhu, Bei Zhong, Shicong Ruan, Jiandong Li, Peidi Ren, Zhihua Ou, Minfeng Xiao, Min Li, Ziqing Deng, Huanzi Zhong, Fuqiang Li, Wen-jing Wang, Yongwei Zhang, Weijun Chen, Shida Zhu, Xun Xu, Xin Jin, Jingxian Zhao, Nanshan Zhong, Wenwei Zhang, Jincun Zhao, Junhua Li, Yonghao Xu

**Affiliations:** ^1^BGI-Shenzhen, Shenzhen, China; ^2^Shenzhen Key Laboratory of Unknown Pathogen Identification, BGI-Shenzhen, Shenzhen, China; ^3^State Key Laboratory of Respiratory Disease, National Clinical Research Center for Respiratory Disease, Guangzhou Institute of Respiratory Health, The First Affiliated Hospital of Guangzhou Medical University, Guangzhou, China; ^4^BGI Education Center, University of Chinese Academy of Sciences, Shenzhen, China; ^5^Institute of Infectious Disease, Guangzhou Eighth People's Hospital of Guangzhou Medical University, Guangzhou, China; ^6^School of Biology and Biological Engineering, South China University of Technology, Guangzhou, China; ^7^Guangdong Provincial Key Laboratory of Biomedical Imaging, Department of Infectious Diseases, Guangdong Provincial Engineering Research Center of Molecular Imaging, The Fifth Affiliated Hospital, Sun Yat-sen University, Zhuhai, China; ^8^The Sixth Affiliated Hospital of Guangzhou Medical University, Qingyuan People's Hospital, Qingyuan, China; ^9^Yangjiang People's Hospital, Yangjiang, China; ^10^Laboratory of Genomics and Molecular Biomedicine, Department of Biology, University of Copenhagen, Copenhagen, Denmark; ^11^Guangdong Provincial Key Laboratory of Human Disease Genomics, Shenzhen Key Laboratory of Genomics, BGI-Shenzhen, Shenzhen, China; ^12^BGI PathoGenesis Pharmaceutical Technology, BGI-Shenzhen, Shenzhen, China; ^13^Shenzhen Engineering Laboratory for Innovative Molecular Diagnostics, BGI-Shenzhen, Shenzhen, China; ^14^Guangdong Provincial Key Laboratory of Genome Read and Write, BGI-Shenzhen, Shenzhen, China

**Keywords:** SARS-CoV-2, population bottleneck, intra-host variation, human to human transmission, evolution

## Abstract

The emergence of the novel human coronavirus, SARS-CoV-2, causes a global COVID-19 (coronavirus disease 2019) pandemic. Here, we have characterized and compared viral populations of SARS-CoV-2 among COVID-19 patients within and across households. Our work showed an active viral replication activity in the human respiratory tract and the co-existence of genetically distinct viruses within the same host. The inter-host comparison among viral populations further revealed a narrow transmission bottleneck between patients from the same households, suggesting a dominated role of stochastic dynamics in both inter-host and intra-host evolutions.

## Author Summary

In this study, we compared SARS-CoV-2 populations of 13 Chinese COVID-19 patients from three hospitals in different cities of Guangdong province. Those viral populations contained a considerable proportion of viral subgenomic messenger RNAs (sgmRNAs), reflecting an active viral replication activity in the respiratory tract tissues. The comparison of identified intra-host variants further showed a low viral genetic distance between intra-household patients and a narrow transmission bottleneck size. Despite the co-existence of genetically distinct viruses within the same host, most intra-host minor variants were not shared between transmission pairs, suggesting a dominated role of stochastic dynamics in both inter-host and intra-host evolutions. Furthermore, the narrow bottleneck and active viral activity in the respiratory tract show that the passage of a small number of virions can cause infection. Our data have therefore delivered a key genomic resource for the SARS-CoV-2 transmission research and enhanced our understanding of the evolutionary dynamics of SARS-CoV-2.

## Introduction

The rapid spread of the novel human coronavirus, SARS-CoV-2, has been causing millions of COVID-19 (coronavirus disease 2019) cases with high mortality rate worldwide (1, 2). As an RNA virus, SARS-CoV-2 mutates frequently (8.5 × 10^−4^ nucleotide substitutions per site per year) during genome replication (3–5), leading to the development of genetically different viruses within the same host. Several studies have reported intra-host single nucleotide variants (iSNVs) in SARS-CoV-2 (7, 8, 25). Recently, we investigated the intra-host evolution of SARS-CoV-2 and revealed genetic differentiation among tissue-specific populations (9). However, it is still not clear how the intra-host variants circulate among individuals. Here, we described and compared viral populations of SARS-CoV-2 among COVID-19 patients within and across households. Our work here demonstrated the utilization of viral genomic information to identify transmission linkage of this virus.

## Results and Discussion

Using both metatranscriptomic and hybrid-capture based techniques, we newly deep sequenced respiratory tract (RT) samples of seven COVID-19 patients in Guangdong, China, including two pairs of patients from the same households, respectively (P03 and P11; P23 and P24). Among the two intra-household pairs, patient P03, P23, and P24 had a travel history to Wuhan city during the early pandemic. The data were then combined with those of 23 RT samples used in our previous study (9), yielding a combined data set of 30 RT samples from 13 COVID-19 patients (Supplementary Table 1).

**Figure 1:**
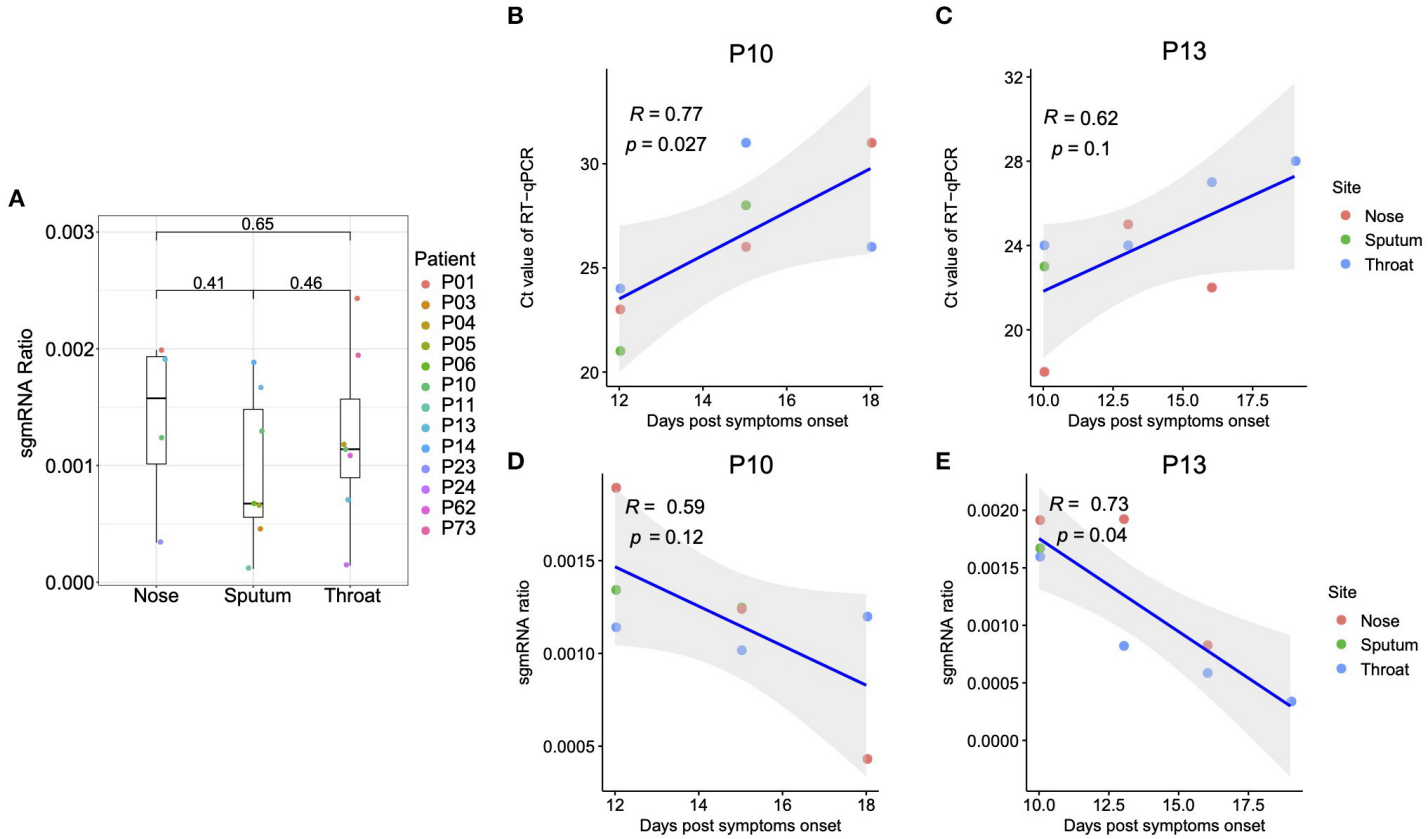
Subgenomic messenger RNAs (sgmRNAs). **(A)** The ratio of sgmRNA of each respiratory sample type (nasal, throat swabs, and sputum). For the patients with multiple samples from the same sample type, only the samples with the median sgmRNA ratio were displayed. **(B,C)** Correlation between the cycle threshold and the days post symptoms onset for patient P10 and P13, respectively. **(D,E)** Correlation between the sgmRNA ratio and the days post symptoms onset for patient P10 and P13, respectively.

A sustained viral population should be supported by an active viral replication (10). We firstly estimated the viral transcription activity within RT samples using viral subgenomic messenger RNAs (**sgmRNAs**), which is only synthesized in infected host cells (11). The sgmRNA abundance was measured as the ratio of short reads spanning the transcription regulatory sequence (TRS) sites to the viral genomic reads (as demonstrated in Supplementary Figure 1). It should be noted that the sgmRNA abundance might be underestimated, given that only the short reads with sufficient length to simultaneously cover both leader and coding flanking regions of the TRS site, which might be improved with long read sequencing in future. Nonetheless, the sgmRNA abundance within nasal and throat swab samples was similar to that within sputum samples (Figure 1A), reflecting an active viral replication in the upper respiratory tract. Notably, the patient P01, who eventually passed away due to COVID-19, showed the highest level of sgmRNA abundance (Supplementary Figure 2). However, due to the limited samples of mild cases, we did not observe a significant difference of sgmRNA abundance between severe and mild cases. For the patients with chronological samples and improved clinical outcomes (P10 and P13), their viral load measured by real-time reverse transcription PCR (qRT-PCR) negatively correlate with the days post symptoms onset with marginal significance (Figures 1B,C). Interestingly, the sgmRNA abundance showed a similar trend across time (Figures 1D,E), reflecting a direct biological association between viral replication and viral shedding in the respiratory tract tissues.

**Figure 2:**
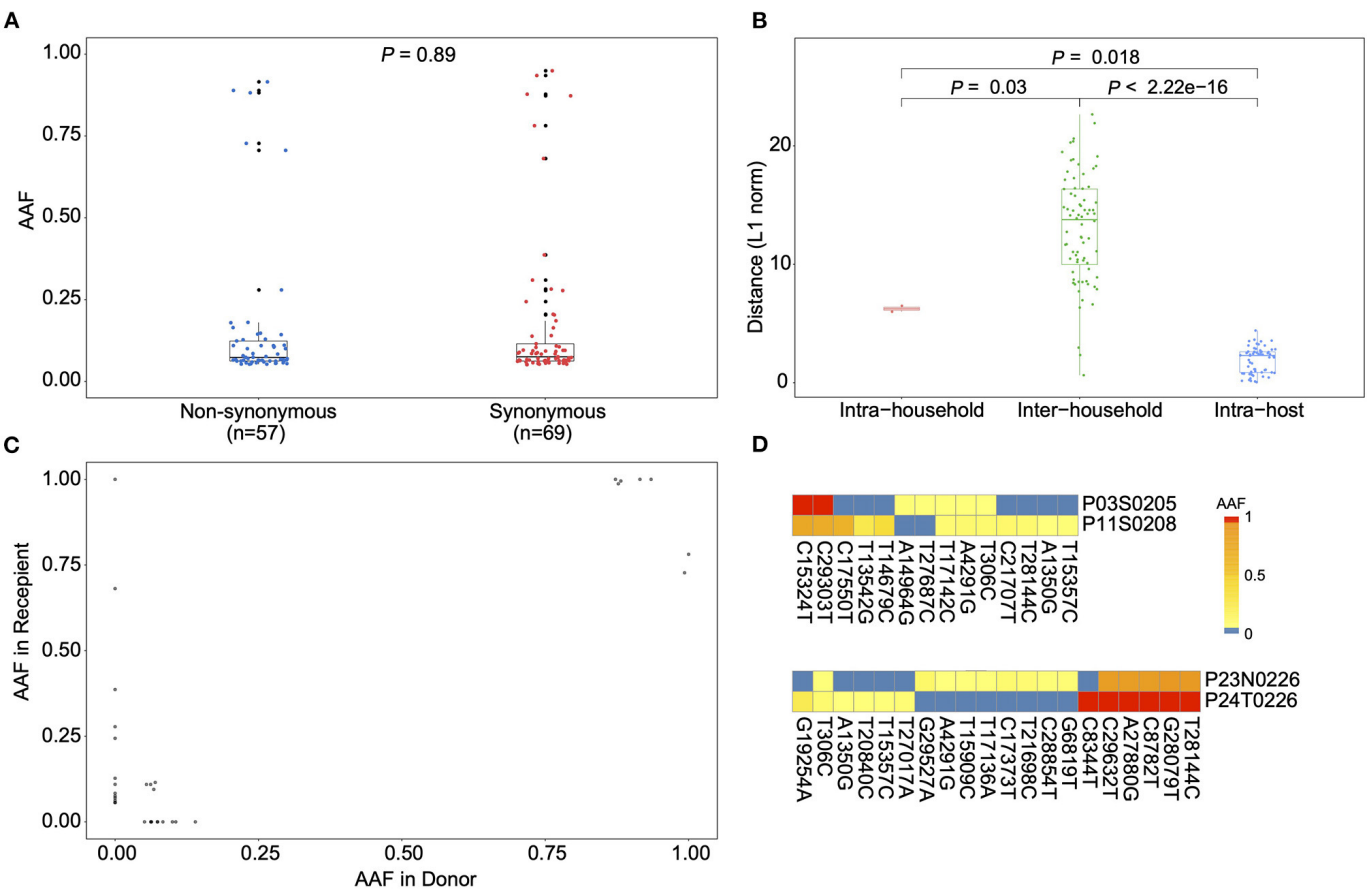
Allele frequency changes of transmission pairs. **(A)** Box plots showing the alternative allele frequency (AAF) distribution of synonymous and non-synonymous intra-host variants. Each dot indicates the median AAF of each iSNV sites of samples from same patient. **(B)** Box plots representing the L1-norm distance distribution among sample pairs. Each dot represents the genetic distance between each sample pair. **(C)** The AAF of donor iSNVs in transmission pairs. Allele frequencies under 5% and over 95% were adjusted to 0% and 1, respectively. **(D)** Heatmap representing the alternative allele frequencies (AAFs) of consensus and intra-host single nucleotide variants (iSNVs) of the two transmission pairs.

Using the metatranscriptomic data, we identified 66 iSNVs in protein encoding regions with the alternative allele frequency (AAF) ranged from 5 to 95% (Supplementary Tables 2, 3; Supplementary Figure 3). Here, an alternative allele was defined as the allele that is different from the allele at the same position of the reference genome. The identified iSNVs showed a high concordance between the AAFs derived from metatranscriptomic and that from hybrid-capture sequences (Spearman's ρ = 0.81, *P* < 2.2e-16; Supplementary Figure 4). We firstly looked for signals of natural selection against intra-host variants. Using the Fisher's exact test, we compared the number of iSNV sites on each codon position against that of the other two positions and detected a marginal but significant difference among them (codon position 1 [*n* = 10, *P* = 0.02], 2 [*n* = 21; *P* = 1], and 3 [*n* = 35; *P* = 0.03]). In contrast to the numbers of iSNV sites, the alternative allele frequency of those iSNVs did not discriminate among the non-synonymous and synonymous categories (Figure 2A), suggesting that most non-synonymous intra-host variants were not under an effective purifying selection within the host. Among the 66 identified iSNVs, 30 were coincided with the consensus variants in the public database as of April 5, 2020 (Supplementary Table 2). Those iSNVs were categorized into common iSNVs, while the iSNVs presented in a single patient were categorized into rare iSNVs. Interestingly, the common iSNVs had a significant higher minor allele frequency compared to the rare iSNVs (Supplementary Figure 5; Wilcoxon rank sum test, *P* = 2.7e-05), suggesting that they may have been developed in earlier strains before the most recent infection.

We then estimated the viral genetic distance among samples in a pairwise manner based on their iSNVs and allele frequencies. The samples were firstly categorized into intra-host pairs (serial samples from the same host), intra-household pairs and inter-household pairs (Figure 2B and Supplementary Table 4). As expected, the intra-host pairs had the lowest genetic distance compared to either intra-household pairs (Wilcoxon rank sum test, *P* = 0.018) and inter-household pairs (Wilcoxon rank sum test, *P* < 2.22e-16). Interestingly, the genetic distance between intra-household pairs was significantly lower than that of inter-household pairs (Figure 2B; Wilcoxon rank sum test, *P* = 0.03), supporting a direct passage of virions among intra-household individuals. Nonetheless, we only observed a small proportion of (3/14 for P03 and P11; 1/20 for P23 and P24) minor intra-host variants shared among intra-household pairs, suggesting that the estimated genetic similarity was mostly determined by consensus nucleotide differences (Figures 2C,D). Based on the AAF of iSNVs in transmission pairs, it seems only the minor virion groups carrying three (from P03) and one variants (from P23) were passed to the recipient, respectively. Specifically, in one intra-household pair (P23 and P24), one patient (P23) contained iSNVs that were coincided with the linked variants, C8782T and T28144C, suggesting that this patient may have been co-infected by genetically distinct viruses. However, the strain carrying 8782C and 28144T was not observed in the intra-household counterpart (P24). Given the small number of COVID-19 cases in Guangdong (about 2,000 total cases), secondary infection from other sources is not likely. Within this intra-household pair, it is likely that there is a narrow transmission bottleneck allowing only the major strain to be circulated, if P23 was infected by all the observed viral strains before the transmission.

The transmission bottlenecks among intra-household pairs were estimated using a beta binomial model, which was designed to allow some temporal stochastic dynamics of viral population in the recipient (12). Here, we defined the donor and recipient within the intra-household pairs according to their dates of the first symptom onset. The estimated bottleneck sizes were 6 (P03 and P11) and 8 (P23 and P24) for the two intra-household pairs (Supplementary Table 5). The observed narrow bottleneck is consistent with two recent studies of SARS-CoV-2 (13, 14). Nonetheless, a loose transmission bottleneck was also observed (8). Similarly, many animal viruses and human respiratory viruses showed a narrow transmission bottleneck (15, 16), while the only study reporting a loose bottleneck among human respiratory viral infections (17) was argued as the generic consequence of shared iSNVs caused by read mapping artifacts (18). The relatively narrow transmission bottleneck sizes are expected to increase the variance of viral variants being circulated between transmission pairs (19). However, given that we can only measure the viral population that were descendants of the founding population, the actual population could have been much larger. Even after successful transmission, virions carrying the minor variants are likely to be purged out due to the frequent stochastic dynamics within the respiratory tract (9), which is also consistent with the low diversity and instable iSNV observed among the RT samples.

The observed narrow transmission bottleneck suggests that, in general, only a few virions successfully enter host cells and eventually cause infection. Although the number of transmitted virions is sparse, they can easily replicate in the respiratory tract, given the observed viral replication activities in all the RT sample types and the high host-cell receptor binding affinity of SARS-CoV-2 (6). The narrow transmission bottleneck also indicate that instant hand hygiene and mask-wearing might be particular effective in blocking the transmission chain of SARS-CoV-2.

In summary, we have characterized and compared SARS-CoV-2 populations of patients within and across households using both metatranscriptomic and hybrid-capture based techniques. Our work showed an active viral replication activity in the human respiratory tract and the co-existence of genetically distinct viruses within the same host. The inter-host comparison among viral populations further revealed a narrow transmission bottleneck between patients from the same households, suggesting a dominated role of stochastic dynamics in both inter-host and intra-host evolution. The present work enhanced our understanding of SARS-CoV-2 virus transmission and shed light on the integration of genomic and epidemiological in the control of this virus.

## Materials and Methods

### Patients

Respiratory tract (RT) samples, including nasal swabs, throat swabs, sputum, were collected from 13 COVID-19 patients during the early outbreak of the pandemic (from January 25 to February 10 of 2020). Those patients were hospitalized at the first affiliated hospital of Guangzhou Medical University (nine patients) in Guangzhou, the fifth affiliated hospital of Sun Yat-sen University (two patients), Qingyuan People's Hospital (1 patient) in Zhuhai and Yangjiang People's Hospital (one patient) Yangjiang. The research plan was assessed and approved by the Ethics Committee of each hospital. All the privacy information was anonymized.

### Dataset Description

Public consensus sequences were downloaded from GISAID on April 5, 2020.

### Sample Preparation and Sequencing

RNA was extracted from the clinical RT samples using QIAamp Viral RNA Mini Kit (Qiagen, Hilden, Germany), which was then tested for SARS-CoV-2 using qRT-PCR. Human DNA was removed using DNase I and RNA concentration was measured using Qubit RNA HS Assay Kit (Thermo Fisher Scientific, Waltham, MA, USA). After human DNA-depletion, the samples were RNA purified and then subjected to double-stranded DNA library construction using the MGIEasy RNA Library preparation reagent set (MGI, Shenzhen, China) following the method used in the previous study (20). Possible environmental or cross contamination during library preparation was tracked using the control RNA samples from human breast cell lines (Michigan Cancer Foundation-7). The constructed libraries were converted to DNA nanoballs (DNBs) and then sequenced on the DNBSEQ-T7 platform (MGI, Shenzhen, China), generating paired-end short reads with 100 bp in length. Most samples were also sequenced using hybrid capture-based enrichment approach that was described in previous study (20). Briefly, the SARS-CoV-2 genomic content was enriched from the double-stranded DNA libraries using the 2019-nCoVirus DNA/RNA Capture Panel (BOKE, Jiangsu, China). The enriched SARS-CoV-2 genomic contents were converted to DNBs and then sequenced on the MGISEQ-2000 platform, generating paired-end short reads with 100 bp in length.

### Data Filtering

Read data from both metatranscriptomic and hybrid capture based sequencing were filtered following the steps described in the previous research (20). Short read data were mapped to a database that contains all the available reference genomes of coronaviridae (including SARS, SARS-CoV-2 and MERS genomes from GISAID, NCBI and CNGB) using Kraken v0.10.5 with default parameters. Low-quality, adaptor contaminations, duplications within the mapped reads were removed using fastp v0.19.5 and SOAPnuke v1.5.6. Low-complexity reads were then filtered using PRINSEQ v0.20.4.

### Profiling of Subgenomic Messenger RNAs (SgmRNAs)

Coronaviridae-like short reads were mapped to the reference genome (EPI_ISL_402119) using the aligner HISAT2 (21). Reads spanning the transcription regulatory sequence (TRS) sites of both leader region and the coding genes (S gene, ORF3a, 6, 7a, 8, E, M, and N gene) were selected to represent the sgmRNAs. The junction sites were predicted using RegTools junctions extract (22). The ratio of sgmRNA reads to the viral genomic RNA reads (sgmRNA ratio) was used to estimate the relative transcription activity of SARS-CoV-2. The sgmRNA ratio and its correlation with days post the first symptom were plotted using the R package *ggplot* (v.3.3.0). To avoid oversampling, for the patients with more than one sample, only the median sgmRNA ratio from samples of that patient was used for comparison among patients.

### Detection of Intra-Host Variants

We defined an intra-host single nucleotide variant (iSNV) as the co-existence of an alternative allele and the reference allele at the same genomic position within the same sample. To identify iSNV sites, paired-end metatranscriptomic coronaviridae-like short read data were mapped to the reference genome (EPI_ISL_402119) using BWA aln (v.0.7.16) with default parameters (23). The duplicated reads were detected and marked using Picard MarkDuplicates (v. 2.10.10) (http://broadinstitute.github.io/picard). Nucleotide composition of each genomic position was characterized from the read mapping results using pysamstats (v. 1.1.2) (https://github.com/alimanfoo/pysamstats). The variable sites of each sample were identified using the variant caller LoFreq with default filters and the cut-off of 5% minor allele frequency (*n* = 89). After removing variable sites at UTR regions (*n* = 12), the sites with more than one alternative allele (*n* = 0), and the sites with only fixed variants (AAF > 95%) were filtered out (*n* = 9). All the iSNVs with less than five metatranscriptomic reads were verified using the hybrid capture data (at least two reads), and thus removed two iSNV sites. The rest 66 sites were regarded as iSNV sites. The identified iSNVs were then annotated using the SnpEff (v.2.0.5) with default settings (24). Alternative allele frequencies between synonymous and non-synonymous iSNV sites were tested with Wilcoxon rank sum test. Each dot indicates the median AAF of the same iSNV site of samples from same patient. All the plots were visualized using the R package ggplot (v.3.3.0).

### Genetic Distance

The genetic distance between sample pairs was calculated using L1-norm distance, as defined by the following formula. To avoid oversampling, for the patients with more than one sample, only the median AAF among all samples of that patient was used for distance comparison. The L1-norm distance (*D*) between sample pairs is calculated by summing the distance of all the variable loci (*N*). The distance on each variable locus is calculated between vectors (*p* and *q* for each sample) of possible base frequencies (*n* = 4).

$$\begin{array}{l} D=\;\overset{N}{\underset{k=1}{\sum }}\overset{n}{\underset{i=1}{\sum }}|p_{i}-q_{i}| \end{array}$$

To verify the result, L2-norm distance (Euclidean distance) between sample pairs was calculated. The L2-norm distance *d*(*p, q*) between two samples (*p, q*) is the square root of sum of distance across all the variable loci (*N*), as defined by the following formula.

$$\begin{array}{l} d\left(p,q\right)=\sqrt{\overset{n}{\underset{i=1}{\sum }}{\left(p_{i}-q_{i}\right)}^{2}} \end{array}$$

The comparison of genetic distances among sample pair categories was performed using the Wilcoxon rank-sum test.

### Beta Binomial Model of Bottleneck Size Estimation

A beta-binomial model was used to estimate bottleneck sizes between donor and recipient (12) (https://github.com/weissmanlab/BB_bottleneck). The beta-binomial model can estimate the probability of variant being detected in the recipient viral population under the prior condition of founding population, allowing variant frequency changes between founding time and sampling time. Here, the bottleneck size represents the number of virions that pass into the recipient and finally shape the sequenced viral population. The patient with the earlier symptom onset date was defined as the donor, while the other was defined as the recipient. For each variable site, variant frequencies within both donor and recipient, read depth and number of reads supporting the mutation within the recipient were used as input of the beta-binomial model. In this model, the virus transmission from donor to the recipient was regarded as a Bernoulli trial, and the probability of a given number of virions carrying this mutation follows a binomial distribution. The maximum-likelihood estimates (MLE) of bottleneck sizes were estimated within 95% confidence intervals. In our data, we got 6 and 8 virions as the estimated transmission bottleneck size of the two donor-recipient pairs, as the probabilities of their beta-binomial distributions reached maximums, respectively.

## Data Availability Statement

The data that support the findings of this study have been deposited into CNSA (CNGB Sequence Archive) of CNGBdb with the accession number CNP0001111 (https://db.cngb.org/cnsa/).

## Ethics Statement

The studies involving human participants were reviewed and approved by the Ethics Committee of the first affiliated hospital of Guangzhou Medical University, the fifth affiliated hospital of Sun Yat-sen University, Qingyuan People's Hospital and Yangjiang People's Hospital, respectively. Informed consent was obtained from all participants enrolled in the study. All the privacy information was anonymized.

## Author Contributions

DW, YX, JL, WZ, and JZ conceived the study. YW, LZ, and YL collected clinical specimen and executed the experiments. DW, WS, XC, and JJ analyzed the data. DW, YW, and ZZ wrote the manuscript. All the authors participated in discussion and result interpretation and revised and approved the final version.

## Conflict of Interest

The authors declare that the research was conducted in the absence of any commercial or financial relationships that could be construed as a potential conflict of interest.

## Supplementary Material

Supplementary Figure 1 Structure of subgenomic messenger RNAs (sgmRNAs).

Supplementary Figure 2 Transcription profile of subgenomic messenger RNAs (sgmRNAs) of each patient.

Supplementary Figure 3 Heatmap representing the alternative allele frequencies (AAFs) of consensus and intra-host single nucleotide variants (iSNVs) of 30 patients.

Supplementary Figure 4 Concordance between minor alternative allele frequencies (AAFs) derived from metagenomic and hybrid capture data.

Supplementary Figure 5 Alternative allele frequency (AAF) distribution of rare and common iSNVs. Each dot indicates the median AAF of each iSNV sites of samples from same patient.

Supplementary Table 1 Demography and clinical outcomes of COVID-19 patients.

Supplementary Table 2 Summary of iSNVs.

Supplementary Table 3 Frequency of iSNVs.

Supplementary Table 4 Inter-host genetic distance (L1 and L2-norm).

Supplementary Table 5 Bottleneck size of intra-household pairs.
