# Supplementary figures and images for: Population Bottlenecks and Intra-host Evolution During Human-to-Human Transmission of SARS-CoV-2
Source: Front Med (Lausanne). 2021 Feb 15;8:585358. doi: 10.3389/fmed.2021.585358 (PMC7917136; doi:10.3389/fmed.2021.585358)

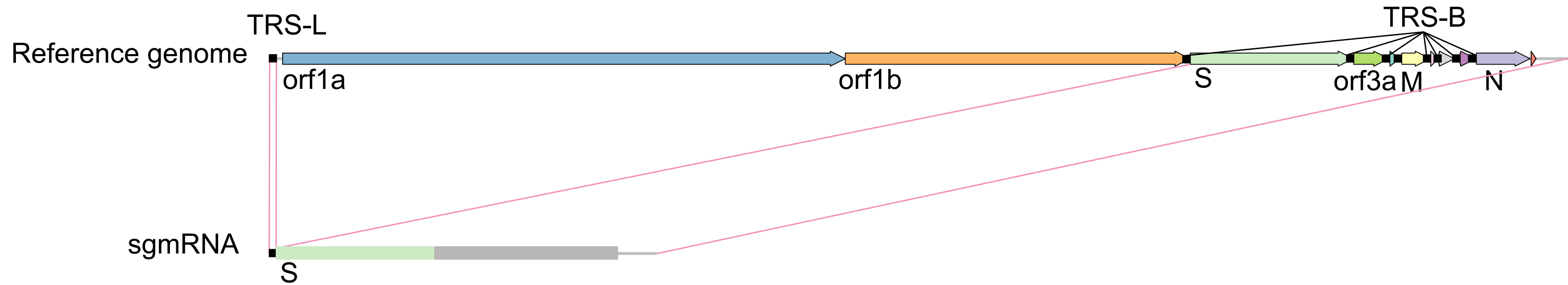

Supplement: Supplementary Figure 1 — Structure of subgenomic messenger RNAs (sgmRNAs). [file Data_Sheet_1.PDF]

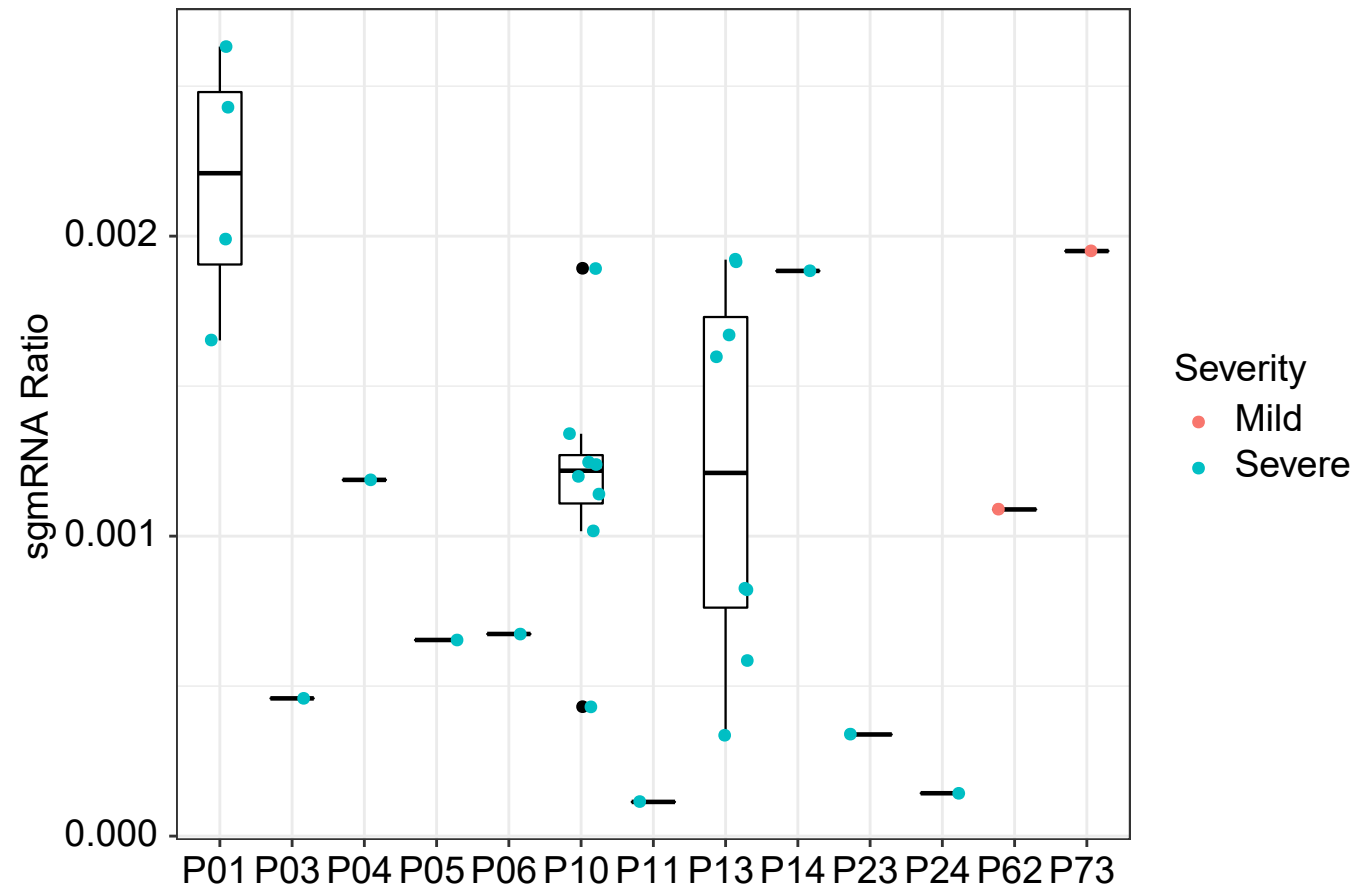

Supplement: Supplementary Figure 2 — Transcription profile of subgenomic messenger RNAs (sgmRNAs) of each patient. [file Data_Sheet_2.PDF]

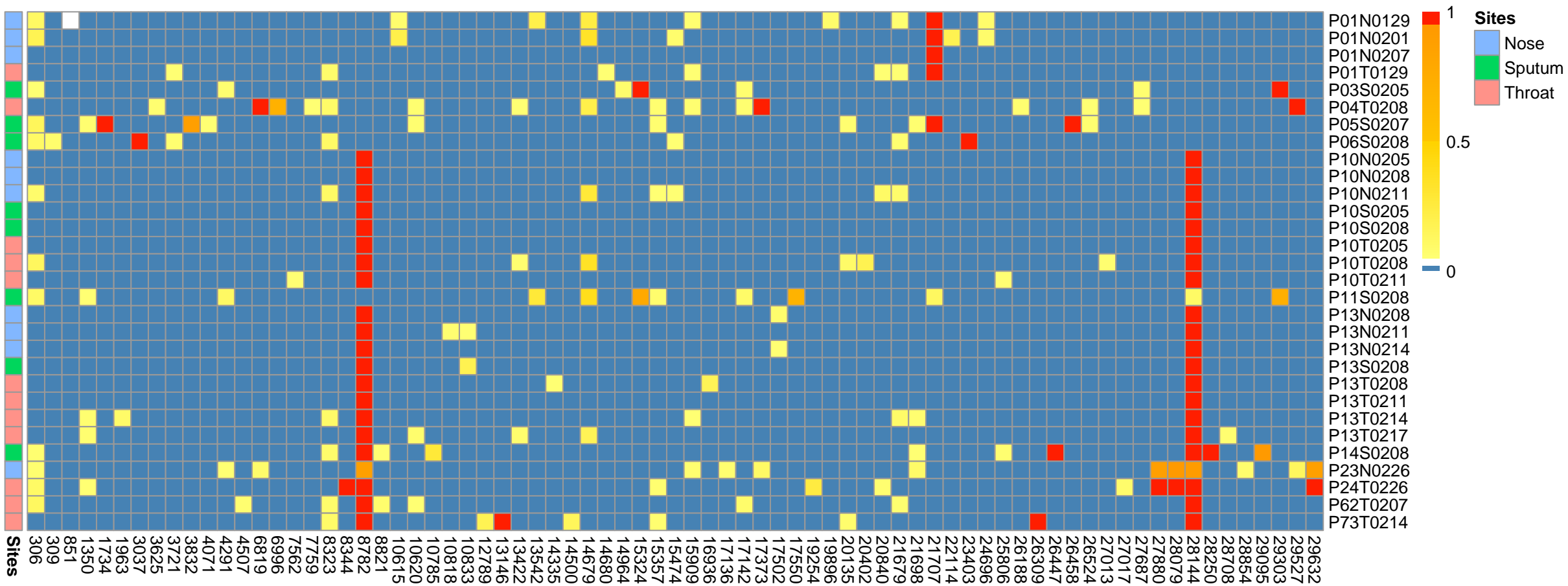

Supplement: Supplementary Figure 3 — Heatmap representing the alternative allele frequencies (AAFs) of consensus and intra-host single nucleotide variants (iSNVs) of 30 patients. [file Data_Sheet_3.PDF]

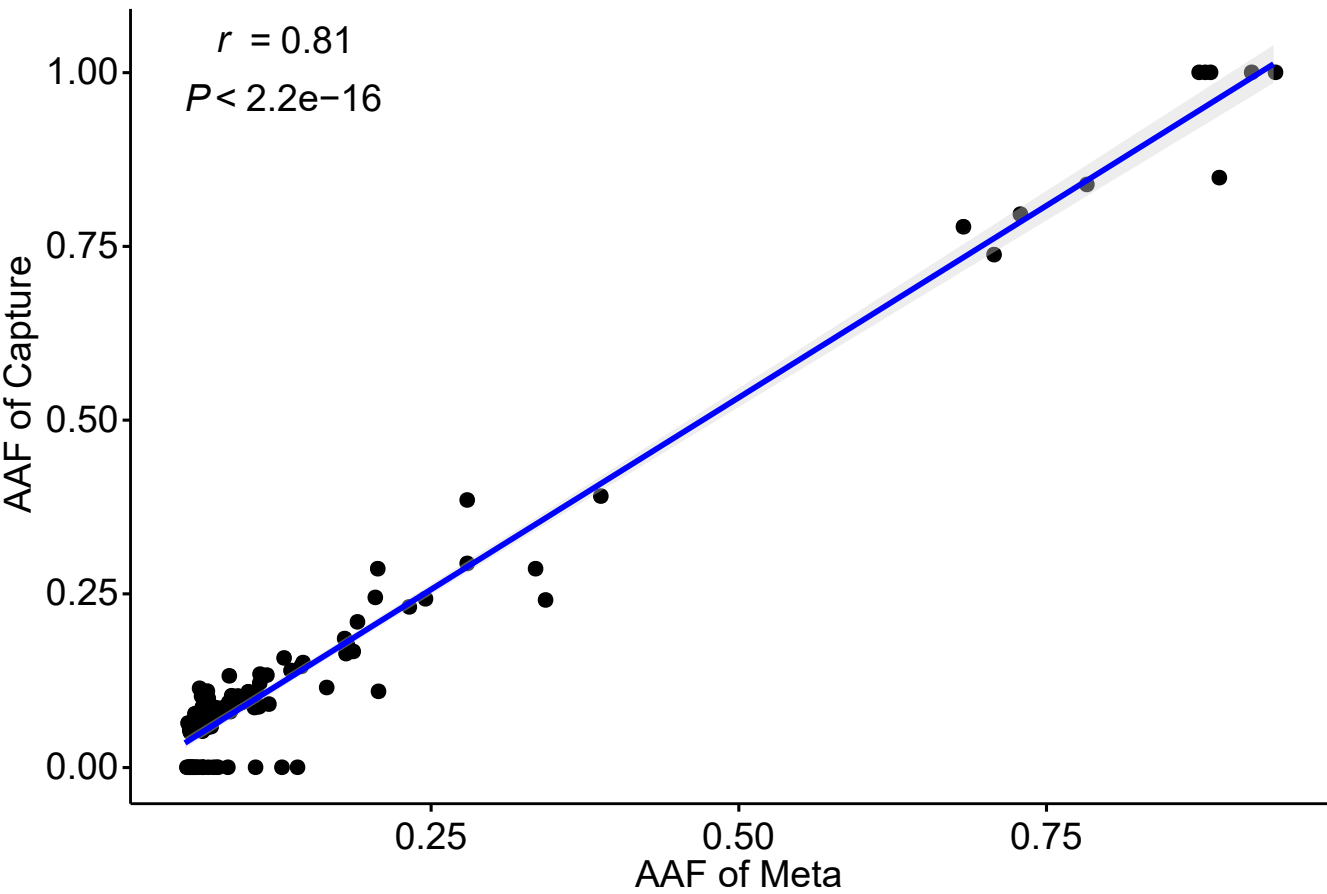

Supplement: Supplementary Figure 4 — Concordance between minor alternative allele frequencies (AAFs) derived from metagenomic and hybrid capture data. [file Data_Sheet_4.PDF]

AAF

Wilcoxon,  $p = 2.7e-05$

Rare iSNV  
(n=80)

Common iSNV  
(n=46)

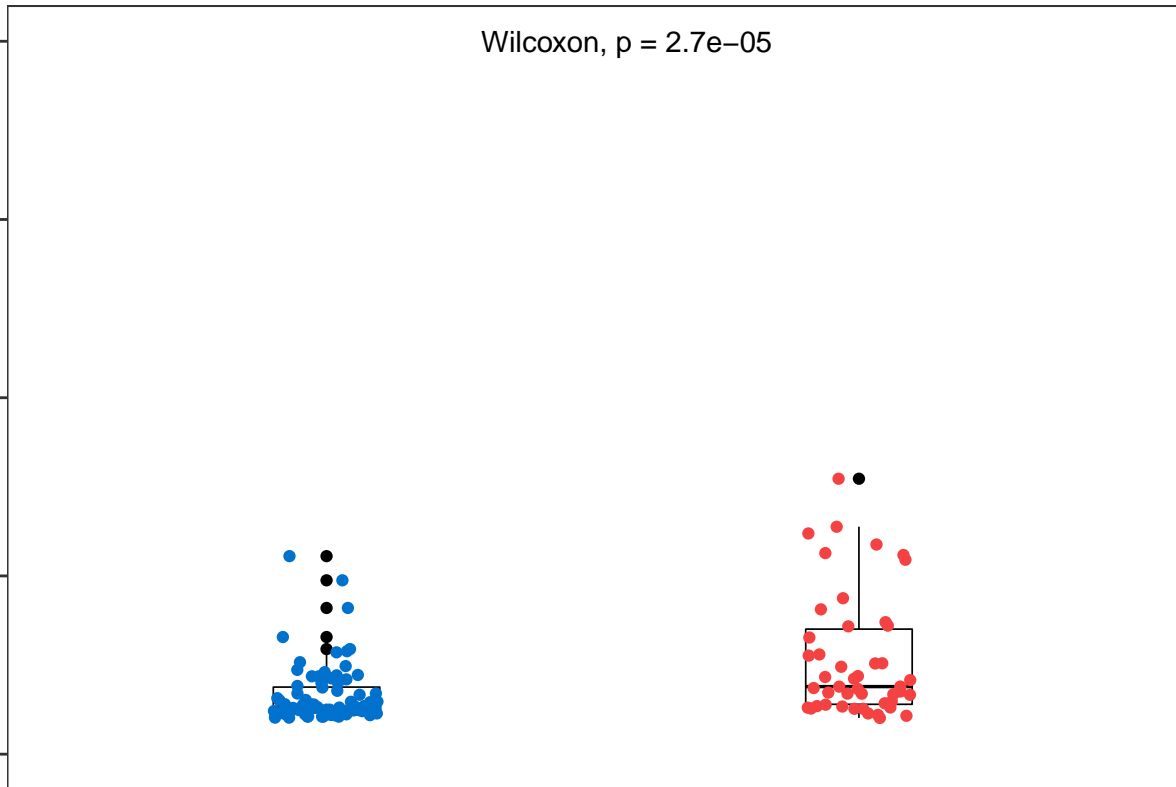

Supplement: Supplementary Figure 5 — Alternative allele frequency (AAF) distribution of rare and common iSNVs. Each dot indicates the median AAF of each iSNV sites of samples from same patient. [file Data_Sheet_5.PDF]
